# Review of the Current State of Freely Accessible Web Tools for the Analysis of 16S rRNA Sequencing of the Gut Microbiome

**DOI:** 10.3390/ijms231810865

**Published:** 2022-09-17

**Authors:** Jerald Conrad Ibal, Yeong-Jun Park, Min-Kyu Park, Jooeun Lee, Min-Chul Kim, Jae-Ho Shin

**Affiliations:** 1 NGS Core Facility, Kyungpook National University, Daehak-ro 80, Daegu 41566, Korea; 2 Department of Applied Biosciences, Kyungpook National University, Daehak-ro 80, Daegu 41566, Korea; 3 Department of Integrative Biotechnology, Kyungpook National University, Daehak-ro 80, Daegu 41566, Korea

**Keywords:** 16S rRNA, gut microbiome, next-generation sequencing, microbiome analysis, web-based tool

## Abstract

Owing to the emergence and improvement of high-throughput technology and the associated reduction in costs, next-generation sequencing (NGS) technology has made large-scale sampling and sequencing possible. With the large volume of data produced, the processing and downstream analysis of data are important for ensuring meaningful results and interpretation. Problems in data analysis may be encountered if researchers have little experience in using programming languages, especially if they are clinicians and beginners in the field. A strategy for solving this problem involves ensuring easy access to commercial software and tools. Here, we observed the current status of free web-based tools for microbiome analysis that can help users analyze and handle microbiome data effortlessly. We limited our search to freely available web-based tools and identified MicrobiomeAnalyst, Mian, gcMeta, VAMPS, and Microbiome Toolbox. We also highlighted the various analyses that each web tool offers, how users can analyze their data using each web tool, and noted some of their limitations. From the abovementioned list, gcMeta, VAMPS, and Microbiome Toolbox had several issues that made the analysis more difficult. Over time, as more data are generated and accessed, more users will analyze microbiome data. Thus, the availability of free and easily accessible web tools can enable the easy use and analysis of microbiome data, especially for those users with less experience in using command-line interfaces.

## 1. Introduction

During the past decade, much attention has been focused on archaea, bacteria, and fungi that form the microbiome owing to the effect they have on health and the environment [1]. A microbiome denotes a set of microorganisms residing in a specific biological niche and includes their genomic content and metabolic products [2,3]. Microbiomes are either host-associated (microorganisms living in organisms, such as humans, other animals, and plants), or free-living (microbial groups found in water and soil) [3]. There has been a sudden shift in our understanding of the crucial role of microbes; from the environment to the human body, it is now widely accepted that microbial communities are the critical components of their ecosystems, aside from the classical view of these entities as mainly infectious pathogens; therefore, the disruption of these communities can be detrimental [4].

“Next-generation sequencing” (NGS) technologies were introduced nearly two decades ago; they transformed biomedical research, resulting in an increase in the sequencing data output [5,6]. With the emergence and dramatic improvement in high-throughput technology and the extreme reduction in the associated costs, NGS technology has made large-scale sampling and sequencing possible, even for individual laboratories. Among the high-throughput sequences obtained is the 16S rRNA gene sequences, which explore the microbial diversity that is relevant to multiple disciplines, ranging from biology and medicine to ecology and environmental sciences. This is because it has been used as a biomarker for archaea and bacteria owing to its conserved regions and relatively short length, which allows for easy sequencing [7]. Multi-omic technology has also promoted collaborative efforts toward a grand vision across the international research community, as demonstrated by the Earth Microbiome Project (EMP) and Human Microbiome Project (HMP) [8,9,10]. The information collected on the human microbiome in recent years is dominated by the data generated through large-scale ventures to characterize the human microbiome, namely the European Metagenomics of the Human Intestinal Tract (MetaHIT) and the NIH-funded Human Microbiome Project (HMP) [11,12]. The data generated through these projects are high in volume and have helped to introduce various interpretations based on a broad range of sources. There has been an increased interest in the human gut microbiome. Until recently, the available literature was insufficient regarding the human gut microbiome to support the development of new strategies for the diagnosis and treatment of diseases. Multiple diseases can arise as a result of the perturbation of the gut microbiome (e.g., irritable bowel syndrome, chronic idiopathic constipation, colorectal cancer, and obesity) [13]. According to a study by Cani [14], approximately 4000 papers associated with the gut microbiota were published in 2017, and more than 12,900 publications have been dedicated to the study of the gut microbiota between 2013 and 2017.

With this large volume of data in mind, the processing and downstream analysis of the data are important to achieve meaningful results and interpretations. The quality of NGS data is also important for various downstream analyses, such as gene expression studies, genome sequence assembly, and microbiome analysis [15,16]. Prior to analysis, the sequencing data must first be checked and processed. The usual protocol is to first assess the quality and depth of the reads [17,18]. Then, most pipelines start by performing quality control on the datasets to increase the accuracy of subsequent processing [16]. Some examples of these preprocessing techniques are the removal of duplicate reads and the deletion of low-quality reads. At present, different tools are available for sequence trimming [19,20]. The next step includes the use of various pipelines to process the NGS data for further downstream analysis, such as mothur [21], Quantitative Insights Into Microbial Ecology (QIIME) [22], and its updated version QIIME2 [23], which have made it easier for scientists to deal with the high volume of data produced from sequencing. Analysis of NGS data is the last step before obtaining final results [24].

**Figure 1:**
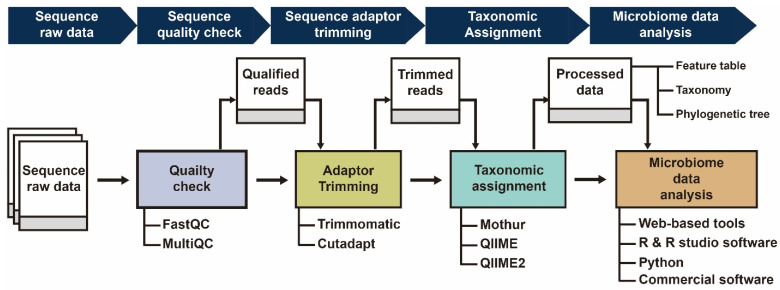
Graphical representation of the overall general workflow for analyzing 16S rRNA gene microbiome data.

Perhaps the most important step of NGS data processing is data analysis and visualization. Novel methods that account for this final step are required for the proper investigation of the microbiome data. Most of the newly developed methods can be employed using Python (e.g., QIIME2; [23]), and R (e.g., the phyloseq package; [25]. Big data analysis has steadily increased due to the availability of NGS data and an increased interest in analyzing microbiome data. An issue may arise if researchers have little experience in using programming languages such as R and python. Although both are incredibly dominant and flexible, learning and getting accustomed to these programming languages can be challenging for beginners (i.e., both clinicians and researchers who only deal in wet-lab experiments). We provide a general workflow for processing microbiome data in Figure 1.

In recent years, the emergence and development of web-based tools have enabled researchers investigating the microbiome to easily perform comprehensive meta-analyses, statistical analyses, and the interactive visualization of microbiome data without any need for previous coding experience [26]. Here, we reviewed and tried to compare a variety of the available open-access web tools and select those that are practical and easy to use when analyzing the human gut microbiome datasets.

## 2. Freely Accessible Web-Based Tools for Microbiome Analysis

### 2.1. Visualization and Analysis of Microbial Population Structures (VAMPS)

Visualization and Analysis of Microbial Population Structure (VAMPS, http://vamps2.mbl.edu, accessed on 10 September 2022), which was developed in 2014, is a free web-based service that offers a range of visualizations and analyses for the interactive and iterative exploration of microbial communities through a comparison of marker gene data. This method uses PHP (v5.2.11) and JavaScript to create the visual front-end and uses Apache (v2.2.25) as its webserver. Additionally, MySQL databases are used for the storage of sequences, taxonomy, and user data.

VAMPS users can start their analysis by uploading the NGS output files, usually using the marker genes (16S rRNA genes for bacterial and archaeal sequences). The VAMPS system can assign the taxonomy for the sequences using oligo-typing, reference-based clustering, species level phylotype (SLP) with average linkage, or UCLUST after filtering the low-quality reads. Otherwise, the users may opt to perform their own quality filtering and taxonomic assignments and upload their data as input using VAMPS analytical tools.

This service can be used with a public account; however, those users who upload their own data are required to have a personal account. Visualization datasets include the most common alpha and beta diversity metrics, and they also contain heatmaps, dendrograms, principal coordinate analysis, bar and pie charts, taxonomy, and operational taxonomic unit (OTU) tables; OTU is the unit used in numerical taxonomy including their unique underlying sequences. These sequences are links to sequence distributions underlying the microbial community, which can be used to cross-check the taxonomy or query the external databases. Another unique feature of VAMPS is its flexibility in taxonomy selection, as users can combine multiple taxonomic levels using taxa-based abundance thresholds for analysis [27].

### 2.2. MicrobiomeAnalyst

MicrobiomeAnalyst (https://www.microbiomeanalyst.ca/, accessed on 10 September 2022) is a web-based program that allows the clinical and scientists who run wet-lab analysis to easily perform exploratory analysis on abundance profiles and taxonomic signatures based on microbiome studies. This program is based on Java, R, and JavaScript. The R phyloseq package [24], in particular, is used for statistical analysis and visualization and for improving computation efficiencies. The system is organized on a Google Cloud server with 32 GB RAM and 8 CPUs (2.6 GHz each).

MicrobiomeAnalyst’s main attributes include the following: (1) It supports an array of common and advanced methods for taxonomic diversity analysis, functional profiling, visualization, and significance testing; (2) it also supports various data filtering and transformation methods, along with well-established, recent algorithms for differential abundance analysis; (3) it features a fully featured metabolic network visualization framework for the intuitive exploration of results from functional profiling; (4) it supports meta-analysis compatible with public datasets for context reference and pattern discovery via 3D visual analytics; (5) it supports enrichment analysis based on more than 300 taxa sets which are manually curated and collected from the literature and public databases. To our knowledge, MicrobiomeAnalyst is still being updated, with the latest being on 08/29/2022. It has been developed by the XiaLab at McGill University (Montreal, Quebec, Canada).

Four modules are involved in MicrobiomeAnalyst. First is the Marker Data Profiling (MDP) module, which is designed for the 16S rRNA marker gene survey data. The second is the Shotgun Data Profiling (SDP) module, which includes the functions for analyzing the metagenomic or metatranscriptomic data. The third is the Taxon Set Enrichment Analysis (TSEA) module, which is designed to identify the biologically or ecologically meaningful patterns in a given list of important taxa. The last one is the Projection with Public Data (PPD) module that allows users to visually compare their data with MicrobiomeAnalyst’s own collection of datasets—in a manner similar to that available with VAMPS—for identifying patterns and new biological insights. MicrobiomeAnalyst uses the outputs from both mothur and QIIME, making use of the OTU table file and the more recently used Biological Observation Matrix (BIOM) file, which stores information on OTUs, taxa, or genes. Chong et al. [26] provide a detailed protocol for the use of the MicrobiomeAnalyst for microbiome analysis. After uploading the required files, the users can choose to filter and normalize their data. Similar to VAMPS, MicrobiomeAnalyst also includes the most common alpha and beta diversity metrics and taxonomic diversity profiling using heatmaps, dendrograms, principal coordinate analysis, and bar and pie charts. In addition, it also allows for the prediction of metabolic potentials and profiling of the functional diversity using the Phylogenetic Investigation of Communities by Reconstruction of Unobserved States (PICRUSt) [28] and Tax4fun [29]. Moreover, a comparative analysis may be performed using MicrobiomeAnalyst, such as differential abundance analysis, which allows users to perform statistical comparisons to identify the significantly different features in OTUs using edgeR [30] and DESeq2 [31]. Biomarker identification and classification may also be achieved using two well-known methods, namely the linear discriminant analysis of size effect (LEfSe), which was developed to help identify robust and biologically significant features for biomarker discovery, and random forest, a non-parametric machine learning algorithm that has performed well in many recent microbiome data analyses and classifications [2,26]. In addition, MicrobiomeAnalyst also provides example datasets using the data from Human Moving Picture, which uses a biom file with a tree [32], Mammalian Gut, which uses the plain text file [33], Mothur output file using Human stool [34], biom file with an aging mouse gut dataset [35], and a plain text file with a tree file using the Pediatric IBD dataset from the Integrative Human Microbiome Project Consortium (iHMP).

### 2.3. Mian

Mian (https://miandata.org/, accessed on 11 September 2022) is an interactive web-based data discovery platform containing a rich set of visualization and machine learning tools to help users examine the microbial community in the context of categorical and numerical sample metadata. The front end of Mian is implemented using HTML5, CSS, and JavaScript/jQuery. Visualizations are rendered using the D3.js and Chart.js libraries. Meanwhile, the backend is implemented using Python 3.6.7, which includes the use of the Flask web framework. Tools such as the beta diversity include R scripts, which have an R 3.6.1 runtime and are integrated with Python using the rpy2 library. The SciPy library is mainly used for statistical testing and NumPy for matrix manipulations. Machine learning tools use either scikit-learn or TensorFlow. The Mian web server is deployed on an AWS Elastic Computing (EC2) m5a.large instance, which has 8GB of RAM and AMD EPYC 7000 series processors clocked at 2.5 GHz, running Ubuntu 16.04. The Amazon Elastic Block Store (EBS) storage is employed for durability and scalability. However, the Mian web server can be successfully deployed in any environment that supports Python and R and has been tested running in both the OSX and SUSE Linux operation systems. It is built at the University of British Columbia and is supported by the Providence Health Care Research Institute and Centre for Heart Lung Innovation at St. Paul’s Hospital, with the latest update from 2021.

Similar to MicrobiomeAnalyst, Mian makes use of the common input file formats (BIOM, CSV/TSV-formatted OTU/ASV tables) generated from Mothur, QIIME, and DADA2. When uploading the data, the users can also opt to normalize using rarefaction, the total and cumulative sum, and upper quartile scaling. In Mian’s case, after uploading the files and finishing the preprocessing, the users can visualize their alpha and beta diversity metrics data using stacked bars, heatmaps, box, donut and scatter plots, PCoA, and NMDS.

Possibly the most unique feature of Mian is its feature selection tools and machine learning algorithms. Unlike MicrobiomeAnalyst, which uses LEfSe for feature selection, Mian uses recursive feature elimination, Fisher’s exact test, and Boruta, which selects the OTUs/ASVs or taxonomic groups that are applied on a random forest classifier and are ideal for selecting all of the groups that are relevant for discriminating between populations, in contrast to finding the non-redundant ones. Moreover, Mian offers the use of machine learning tools to assess the discriminative performance of the taxonomic groups selected through a feature selection tool. Mian uses linear regressor, random forest classifier, and deep learning, which trains a multi-layer perception network on the taxonomic data to predict a numerical or categorical variable. The network can be customized with a different number of fully connected and drop-out layers and a different number of units within each layer [1].

### 2.4. Global Catalogue of Metagenomics (gcMeta)

Global Catalogue of Metagenomics (gcMeta) is a part of the Chinese Academy of Sciences Initiative of Microbiome (CAS-MI) and has two main features: first design and implementation as a standardized and state-of-the-art database management tool for support, long-term preservation, and integrating microbiome projects worldwide, and second, to provide web tools and workflows for massive data analysis (https://gcMeta.wdcm.org/, accessed on 11 September 2022). The system is based on centralized computing and storage resources. Its database management system is separated into metadata, sequence raw data, and user information management. The updated version of gcMeta is constructed on the basis of PostgreSQL for both the metadata and user information and makes use of MongoDB for sequencing the raw data index information. The system is operated on Linux servers, and the web interface was developed through Python.

The platform provides management, analysis, and publication services for microbiome-related data. The analysis tools in gcMeta are installed based on a Docker container which allows users to perform analyses. The users can upload the raw data and metadata to gcMeta’s system through a web submission interface. After checking the quality, the data can be browsed on the system under the user’s account. Although gcMeta provides five main frameworks, we focused only on the use of 16S rRNA analysis. Using the Docker container, the 16S rRNA sequence can be processed using the widely known QIIME2 to produce a feature table and taxonomy. They also make use of QIIME2 for diversity analysis and PICRUSt and biomarker discovery using LEfSe [10].

### 2.5. Microbiome Toolbox

Microbiome Toolbox allows for the exploration and understanding of the identification of key microbiome features to depict an appropriate microbiome. This platform also focuses on analyses of the microbiome, especially for the human gut. Besides visualization and exploration, microbiome trajectories are also implemented using machine learning algorithms, which can help determine the key features for microbiome analysis. The interactive dashboard can be found at https://microbiome-toolbox.herokuapp.com/, accessed 10 September 2022.

The different types of microbiome data, such as the compositional and functional data tables generated from different technologies such as 16S rRNA or shotgun metagenomics, can be used as inputs on the platform. From our list of web tools, perhaps Microbiome Toolbox is the only one dedicated to analyzing the microbiome data that change with time or the data tables that essentially follow the same longitudinal structure of features changing over time, and the toolbox is oriented more toward the analysis of the Early Life Microbiome in infants [36]. Similar to MicrobiomeAnalyst, Microbiome Toolbox also provides an already modified example dataset from both the mouse [37] and the gut microbiome from breastfed infants [38].

## 3. Comparison of the Web Tools Using Gut Microbiome Dataset

**Figure 2:**
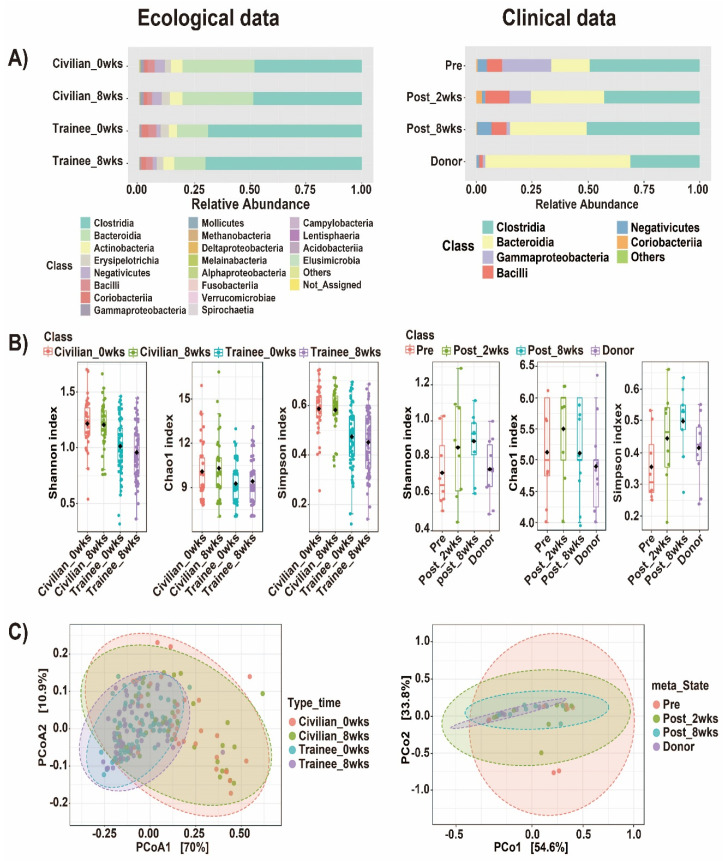
Output figures for (**A**) taxonomic data (class), (**B**), alpha diversity (Shannon, Chao1, and Simpson index), and (**C**) beta diversity (Bray−Curtis dissimilarity) based on different analysis approaches using ecological (**left**) and clinical (**right**) datasets in MicrobiomeAnalyst.

**Figure 3:**
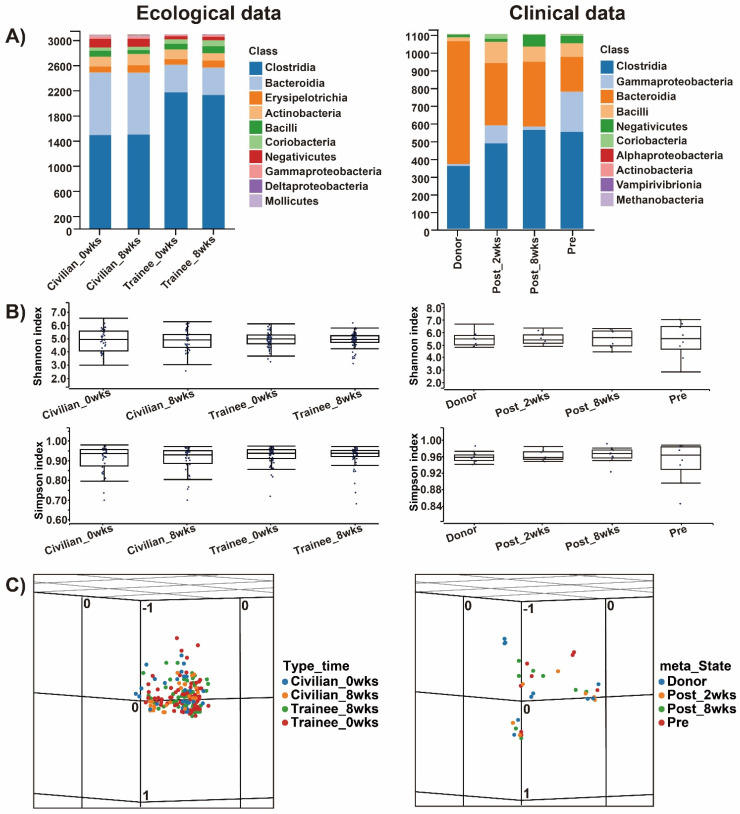
Output figures for (**A**) taxonomic data (class), (**B**), alpha diversity (Shannon, Chao1, and Simpson index), and (**C**) beta diversity (Bray−Curtis dissimilarity) based on different analysis approaches using ecological (**left**) and clinical (**right**) datasets in Mian.

In recent years, interest in studying the gut microbiome has increased and incredibly large volumes of data are being produced and analyzed. NGS sequencing has revolutionized the field of microbiology. It has provided researchers with a cost-effective technology to sequence millions of base pairs and replaces the conventional characterization of bacteria or pathogens through morphology or cultivation-based approaches. It can also be used to interrogate full genomes or exomes to discover novel mutations and disease-causing genes. In the context of microbiome research, it provides a comparative insight into the phylogenetic structure of microbial communities and their potential interactions with the host [39]. In this review, we specifically used those datasets corresponding to the gut microbiome to compare different free web tools for analyzing 16S rRNA gene sequences. We looked at common and basic analyses, such as the alpha and beta diversities, in addition to comparing the unique features that the web tools offer with respect to their usefulness for carrying out gut microbiome analysis. We used two datasets: (1) a clinical dataset, wherein the gut microbiome was analyzed to check the efficacy of fecal microbial transplant (FMT) for people infected with *Clostridioides difficile* [40], and (2) a dataset that uses an ecological analysis approach, wherein the lifestyle factors affecting the gut microbiome of Korean navy trainees [41] are included, for testing these web tools. During the writing of this review, we encountered challenges in using gcMeta, VAMPS, and Microbiome Toolbox due to file format issues and unresponsive web pages and thus proceeded to only use MicrobiomeAnalyst and Mian. As regards gcMeta, an unresponsive page was encountered when trying to create an account or log in, which ultimately resulted in the failure of data upload and analysis. Meanwhile, in terms of VAMPS, the fastq files first need to be formatted in accordance with their algorithm. However, this poses a problem to those users who have a large number of datasets in which time is needed to correct the format of the abovementioned file. The same issue is seen when using Microbiome Toolbox. We believe that the file format issues will result in limited use for the new users who are not familiar with editing the output from the preprocessed data. First, the datasets were processed using QIIME2, and then, the original methods for producing the biome file were used. The files were then inputted into the abovementioned web tools using the default parameters to check the taxa (class level) and alpha and beta diversities (Bray–Curtis dissimilarity); Figure 2 and Figure 3 show the output figures for MicrobiomeAnalyst and Mian, respectively. We also proceeded to use MicrobiomeAnalyst and Mian’s “unique tools”. Although the analysis performed in this review is outside the scope of the original studies, we still explored the different analysis types that the two web tools offer, and the corresponding results are summarized in Table 1.

A comparison of Mian and MicrobiomeAnalyst revealed that both are easy to access, and both have rapid visualization and computation time; both possess options to change the parameters for visualization, although Mian provides fewer options than MicrobiomeAnalyst. However, MicrobiomeAnalyst offers more downstream analysis features than Mian, such as the ability to integrate the predicted functional genes using PICRUSt and Tax4Fun (for bacteria and fungi, respectively). Several studies have used PICRUSt to identify the functional genes present in the gut microbiome [42,43,44]. Bahr et al. [42] used PICRUSt to observe the changes in the gut microbiota of children with atypical antipsychotic risperidone (RSP), while Yun et al. [43] looked at genes in a Korean cohort in the context of how the genes differed based on the body mass index in normal, overweight, and obese individuals. The PICRUSt module of MicrobiomeAnalyst provides users with more options for analyzing their data in detail without having to process the same in a command line interface.

Both Mian and MicrobiomeAnalyst offer the LEfSe analysis, which is mostly used to identify the specific taxa for biomarkers [45,46]. Studies ranging from clinical use, such as examining microbial dysbiosis, which revealed significant differences in bacterial abundances between the healthy controls and colorectal adenoma or intramucosal colorectal carcinoma patients [47], to finding the differences in the gut microbiota between native Tibetan and Han populations through the abundant taxa present [48].

In recent years, there has been an increased use of machine learning to generate models using microbiome data. Machine learning techniques offer a means to analyze high-dimensional data and may be used to reveal the relationships between microbial taxa and environmental features [49,50,51,52]. Mian and MicrobiomeAnalyst provide users with the machine learning algorithm, i.e., random forests, which are arguably the most effective machine learning model for analyzing microbiome data, owing to its high accuracy with respect to classification. It has been verified with a variety of 16S rRNA datasets for the identification of body habitat, host, and disease states [49,50]. Aryal et al. [53] used a random forest for the diagnostic screening of cardiovascular disease using the gut microbiome, while Ai et al. [54] used this model for identifying the gut microbes associated with colorectal cancer. Mian also offers deep learning via a deep neural network employing classification or regression, which is extremely useful because of its flexibility and ability to resolve non-linear cases [55].

**Figure 4:**
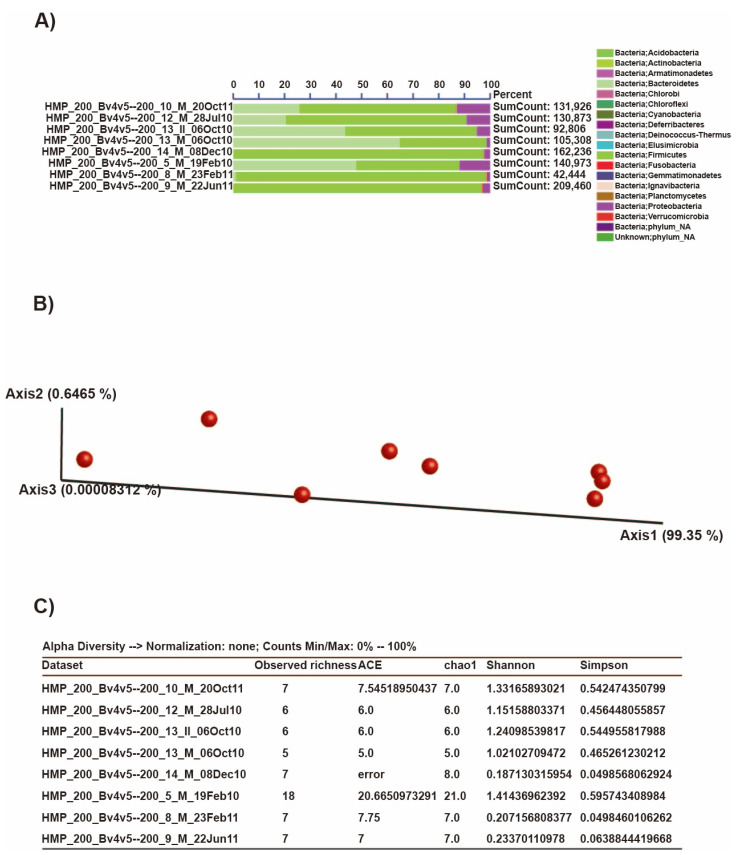
Output figures for (**A**) taxonomic data (phyla), (**B**) beta diversity (Bray–Curtis dissimilarity), and (**C**) alpha diversity (Observed richness, Ace, Chao1, Shannon, and Simpson indices) using the HMP_200 dataset that is readily available in VAMPS.

**Figure 5:**
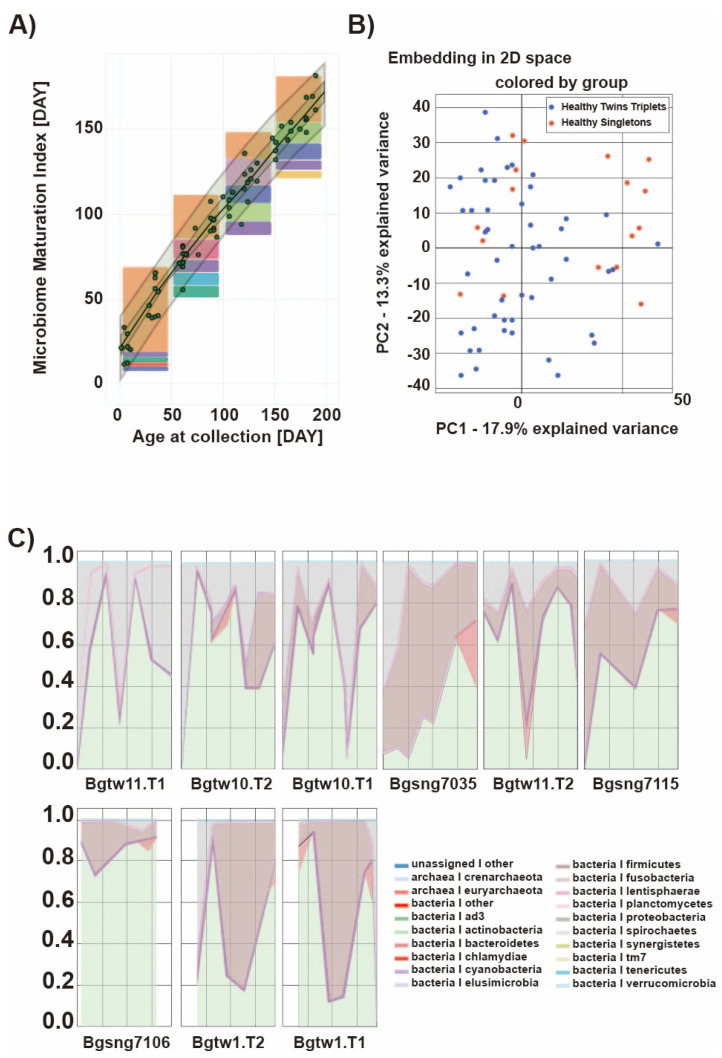
Output figures depicting the (**A**) important features for time trajectory, (**B**) PCA in 2D, and (**C**) dense longitudinal data corresponding to abundance generated using the human gut dataset found in Microbiome Toolbox.

As there were issues with the file format and data curation on VAMPS and Microbiome Toolbox, we opted to use the data that were available within their servers to show what these web tools offer. In the case of VAMPS—using its search engine with the human–gut environment as the source—we found and used the human data HMP_200 (V4–V5 region), which were uploaded to the system between 2010 and 2011. In Figure 4, we show how the taxa and alpha and beta diversities are visualized on VAMPS. In a similar fashion, we also used the sample data corresponding to the human gut microbiome that are readily available in the Microbiome Toolbox’s system (Figure 5), where machine learning algorithms are used to predict the microbiome maturation index through time, in addition to identifying outliers and selecting the key bacteria that are important within the given time trajectory.

### Limitations of Web-Based Tools for Microbiome Analysis

In data analysis using web-based tools, two factors are recognized as important. First is the accessibility of web-based tools. Therefore, we checked the accessibilities of all the web-based tools in this study using a microbiome dataset and found that Mian and MicrobiomeAnalyst, and VAMPS were easily accessible. Conversely, gcMeta and Microbiome Toolbox showed a non-responsive page when logging in, and slow response when uploading the data, respectively. Moreover, an easily input file format for the web-based tool is also important. The methods of the input file format change for Mian and MicrobiomeAnalyst are demonstrated clearly. However, the information regarding an input file format for Microbiome Toolbox and VAMPS is not described in detail. Although those with the knowledge of manipulating input files can access both freely, those with little experience might encounter difficulties when using VAMPS and Microbiome Toolbox.

Generally, statistical methods are chosen based on the distribution (normal or not) and variance (equal or not) of the dataset. In the microbiome data, statistical analysis can emphasize a meaningful microbiome result [56]. We acknowledge that the web-based tools mentioned have different purposes with respect to analyzing the microbiome data. Usually, it is better for the users if different statistical methods are already included in the web tool. We found that statistical analyses are easy to perform using Mian and MicrobiomeAnalyst. Meanwhile, VAMPS was better at visualization rather than statistical analysis in comparison with Microbiome Toolbox and was more efficient at microbiome feature prediction over time.

Taken together, the analysis tools that are included in VAMPS, Microbiome Toolbox, Mian, and MicrobiomeAnalyst offer users a variety of options for easily managing their microbiome data for further downstream analysis.

## 4. Conclusions

In this study, we explored different freely available web-based tools for microbiome analysis using the gut microbiome datasets. Though there is software for analyzing the microbiome data such as CLC Genomics Workbench (QIAGEN, Hilden, Germany), we specifically focused on those tools that are freely accessible. Multiple tools are available for microbiome analysis, such as the R-based Genepiper [57], MANTA [58], and Microbiome Modeling Toolbox [59]—to name a few—but we only focused on web-based tools. In our search, we found VAMPS, MicrobiomeAnalyst, Mian, gcMeta, and Microbiome Toolbox. The abovementioned web tools are all freely accessible; however, there are log-in problems with gcMeta. Similarly, VAMPS and Microbiome Toolbox require an extension for processing the data on their site. Thus, we were left with MicrobiomeAnalyst and Mian, and we compared the analysis tools they offer by evaluating the gut microbiome datasets corresponding to clinical and ecological approaches using the basic analysis employed for the microbiome data (alpha and beta diversities). In the case of MicrobiomeAnalyst and Mian, we also tried to search for other analysis tools that can be used for further downstream analysis. The ability of both web tools to perform different statistical analyses greatly helps in discerning meaningful differences in user data. Moreover, the availability of PICRUSt in MicrobiomeAnalyst provides users with the freedom to analyze the functional genes in their dataset. While the two web-based tools also include LEfSe and random forest models for the selection of biomarkers, Mian provides users with access to deep learning, i.e., using the deep neural network to build a sophisticated model, which can also be used for classification. Collectively, we believe that free web-based tools will allow users, especially clinicians and those new in the field, to make an easier and more practical and refined analysis of the human gut microbiome data.

## Figures and Tables

**Table 1:** Comparison of web-based microbiome tools.

Data Upload and Function		VAMPS	MicrobiomeAnalyst	Mian	gcMeta	Microbiome Toolbox
File format		Edited FASTA	BIOM	BIOM		Edited OTU table
Database		SILVA, Greengenes	SILVA, Greengenes	NA		NA
Common analysis	Rarefaction curve	X	O	O		X
	Bar/stack analysis	O	O	O		X
	Pie chart	O	O	X		X
	Core microbiome analysis	X	O	X		X
	Phylogenetic tree	O	O	X		X
α-Diversity	Shannon index	O	O	O		X
	Simpson index	O	O	O		X
	Richness index	X	O	X		X
	Chao1 index	O	O	X		X
	ACE index	O	X	X		X
	Evenness index	X	X	X		X
β-Diversity	Bray-Custis dissimilarity	O	O	O		O
	Jaccard distance	X	O	X		X
	Unweighted UniFrac	X	O	O		X
	Weighted UniFrac	X	O	O		X
	NMDS	X	O	O		X
	CCA analysis	X	O	X		X
	RDA analysis	X	O	X		X
Correlation and Clustering analysis	Heatmap	O	O	O		O
	Correlation plot	O	O	O		O
	DEseq2	X	O	X		X
	Network analysis	X	O	X		X
Functional gene prediction	PICRUSt	X	O	X		X
	Tax4Fun	X	O	X		X
Comparative analysis	LEfSe	X	O	O		X
	Random Forest	X	O	O		X

O signifies the analysis method is available in the web tool; X signifies the analysis method is not available in the web tool; NA signifies that databases are not provided for phylogenetic analysis; Tools that were currently not accessible were left blank.

## Data Availability

Not Applicable.
